# Nanoionics from Biological to Artificial Systems: An Alternative Beyond Nanoelectronics

**DOI:** 10.1002/advs.202200534

**Published:** 2022-06-16

**Authors:** Jianrui Zhang, Wenchao Liu, Jiqing Dai, Kai Xiao

**Affiliations:** ^1^ Department of Biomedical Engineering Southern University of Science and Technology (SUSTech) Shenzhen 518055 P. R. China; ^2^ Guangdong Provincial Key Laboratory of Advanced Biomaterials Southern University of Science and Technology Shenzhen 518055 P. R. China

**Keywords:** ion transport, ionotronics, nanoelectronics, nanofluidic, nanoionics

## Abstract

Ion transport under nanoconfined spaces is a ubiquitous phenomenon in nature and plays an important role in the energy conversion and signal transduction processes of both biological and artificial systems. Unlike the free diffusion in continuum media, anomalous behaviors of ions are often observed in nanostructured systems, which is governed by the complex interplay between various interfacial interactions. Conventionally, nanoionics mainly refers to the study of ion transport in solid‐state nanosystems. In this review, to extent this concept is proposed and a new framework to understand the phenomena, mechanism, methodology, and application associated with ion transport at the nanoscale is put forward. Specifically, here nanoionics is summarized into three categories, i.e., biological, artificial, and hybrid, and discussed the characteristics of each system. Compared with nanoelectronics, nanoionics is an emerging research field with many theoretical and practical challenges. With this forward‐looking perspective, it is hoped that nanoionics can attract increasing attention and find wide range of applications as nanoelectronics.

## Graphical abstract

Nanoionics, as an analogy of nanoelectronics, refers to the transport phenomena, functions, and applications of ions that are confined in the nanospace. This review introduces the concept, recent remarkable advances, and insight on the current challenges of nanoionics, which can be categorized into biological nanoionics, artificial nanoionics, and hybrid nanoionics.

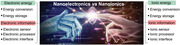

## 1 Introduction

The advancement of nanotechnology over the past several decades has allowed the engineering of material structures across multiple length scales.^[^ ^1^, ^2^ ^]^ The capability of designing multifunctional materials and devices tailored to specific needs has contributed to the great success of the nanoelectronics field.^[^ ^3^ ^]^ Based on semiconductor physics, nanoelectronics has become a crucial part of electronics and is of predominant significance in facilitating human technological progress, particularly in the information technology and energy fields. However, as the dimension decreased to sub‐5 nm scale, higher‐performance solid‐state nanoelectronic devices face physical limitations and performance challenges. For example, the speed and energy efficiency of silicon complementary metal–oxide–semiconductor (CMOS)‐based computing hardware are approaching to its theoretical limit.^[^ ^4^ ^]^ Meanwhile, it is widely believed that nanoelectronics based devices could hardly be comparable to the human brain in terms of high‐level information processing, such as pattern recognition and intelligent reasoning, especially considering the energy efficiency. One of the major reasons lies in the distinct working principle of the communication media between solid‐state nanoelectronics and soft biological nanoionics systems. Cell‐to‐cell communications in an intelligent organism is expressed in the language of various ions and small molecules, in contrast to the mere flow of electrons. Motivated by this observation, the utilization of ions as the charge carriers has emerged as a new research frontier, and received increasing attention.^[^ ^5^, ^6^, ^7^ ^]^

In Nature, the transmission and processing of information, as well as the energy conversation and storage are often mediated and regulated using ions and fluids transportation at small scales.^[^ ^8^ ^]^ An organism is a typical and ideal model of ionic circuits whose function is based on the interplay between pre‐designed structures of biomacromolecules and various ions of different charge, size, and mobility. Taking the inspiration from organisms, the new generation of artificial intelligence systems, including sensory devices, energy devices, and human–machine interfaces that work in a nanoionic way, would possess several key advantages. For example, they could have much lower power consumption than conventional processors, and exploit integrated nonvolatile memory using biocompatible or biodegradable logic circuits for sensing. Additionally, these devices could be explicitly designed to support dynamic learning of complex and unstructured data.^[^ ^9^, ^10^, ^11^ ^]^ Moreover, as exemplified inbatteries and fuel cells, ions are also important for the transformation of chemical energy to electrical energy.^[^ ^12^, ^13^, ^14^ ^]^ Therefore, the significance of ionic charge carriers is by no means less than that of electronic charge carriers.

In recent years, research using ions as charge carriers has received increasing attention in several specific research fields, e.g., electrochemical energy conversion and information storage.^[^ ^15^, ^16^ ^]^ Beyond that, it is believed that ionic charge carriers have broad applications, especially in designing information processors, thanks to the specific characteristics of ions, such as valences, sizes, and polarizabilities. For example, various ionic flows including Na^+^, K^+^, and Ca^2+^, determine electrophysiology in biology.^[^ ^17^, ^18^, ^19^ ^]^ As a result, like biological intelligence organisms, artificial systems that run on ions should have the potential to be more efficient than today's electronic devices. Nanoionics based on multi‐ionic carriers, analogous to nanoelectronics, can emulate biological functionality more directly and can also be used as an emerging technology that bridges artificial intelligence systems and biological systems seamlessly.^[^ ^10^, ^20^, ^21^ ^]^

**Figure 1:**
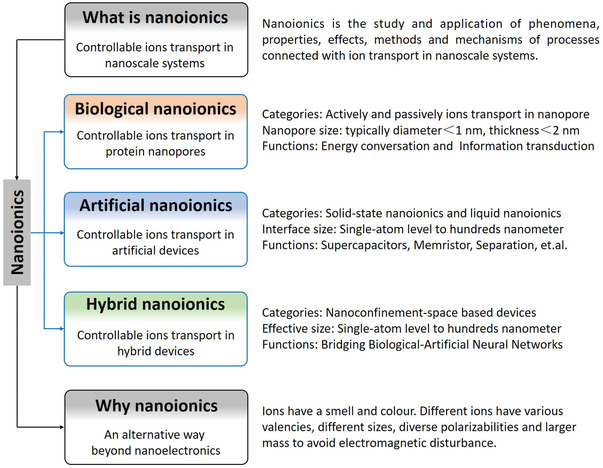
Schematic outline of this review. The first part will give a brief introduction of “what is nanoionics,” following with three main nanoionics systems: biological nanoionics, artificial nanoionics, and hybrid nanoionics. In the last part, we try to answer the question of “why nanoionics” and give a prospect and outlook of nanoionics.

As outlined in **Figure** **1**, herein we summarize the nanoionics systems and structure the field into biological, artificial, and hybrid categories. In the first section, the general concept of nanoionics is illustrated and evolved from conventional notion, and then the three key nanoionics systems – biological, artificial, and hybrid – are summarized with each features enumerated. Finally, a perspective on challenges and future opportunities in the field is charted. Over the course of the entire review, we will rationalize the need of nanoionics as an alternative beyond nanoelectronics. In view of the organization structure and multidisciplinary research background of this topic, we note that this review is not meant to be comprehensive. We will, to the best of our knowledge, direct interested readers to relevant references so that the breadth and depth of the context of this emerging field can be readily appreciated.

## 2 What is Nanoionics?

**Figure 2:**
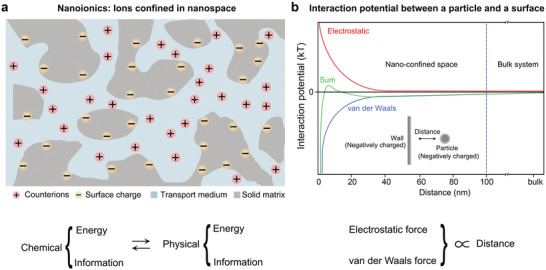
The concept of nanoionics. a) The term nanoionics is to study ion transport in nanoconfined space. The application of nanoionics mainly involves in the transformation of chemical energy or information into electrical energy or information (or vice versa). b) Interaction potential between a particle and a surface (both negatively charged): Both the vdW forces and electrostatic forces are inversely proportional to the distance between surface and particle. The nanoparticle is attracted to the surface by vdW forces (blue line), but repelled by electrostatic forces (red line), and is shown here at the minimum of the combined potential (green line).

As reflected in the nomenclature, the term “nano”‐“ionics” is the study of mechanisms, phenomena, properties, effects, methods, and applications of processes connected with ion transport in nanoconfinement. We emphasize that nanoionics is by no means a new concept or term. The term and conception of nanoionics were first introduced in 1992.^[^ ^22^ ^]^ Later, Maier and several other scientists invested enormous efforts to shape this research topic and made a significant contribution to it.^[^ ^7^, ^23^, ^24^, ^25^ ^]^ Originally, the concept of nanoionics referred to the study of ion transport in all‐solid‐state nanoscale systems. However, nanoionics is also related to ion‐transport and storage phenomena in liquids; therefore, the concepts of nanoionics should be much broader and more general. For instance, the materials of choice should not be restricted to nanostructured solid‐state materials, rather they should be generalized to systems, such as liquid and biological materials, as well as the hybrid of the two. Similarly, the application of nanoionics may not be restricted to typical electrochemistry‐related fields, but also include processes such as energy and information transfer (**Figure** **2**a).

For nanoionics studies in both solid‐state or liquid states, the characteristics of nanostructured materials, such as pore sizes, chemical and physical nature are of pivotal importance for both fundamental understanding and exploration of potential applications. While the effect is not significant in bulk systems, at the nanometer regime, interfacial effects will prevail in controlling ion transport.^[^ ^26^, ^27^ ^]^ In interfacially controlled structures and materials, the overall effect of the interfacial regions increases with decreasing size between two different surfaces, e.g., solid/solid and solid/liquid interfaces. As an example, for the interaction potential between a particle and a surface, both the van der Waals (vdW) and electrostatic forces increase with decreasing distance between the surface and the particle (Figure 2b). In other words, interfacial effects will play a much more crucial role for ion transport in short distances and small volumes.

The interfacial effects are so strong that ion transport in nanoscale space can exhibit anomalous phenomena compared with that in bulk systems.^[^ ^6^, ^28^ ^]^ Spatial limitation could constrain the motion of ions and/or modify their interactions with each other as well as with the nanoconfinement surface. If the ions are placed in a solid‐state nanometer regime, the interfaces are so closely spaced that their effects on ion transport can become significant if not predominant.^[^ ^27^ ^]^ If the ions are surrounded by solvent molecules, they can strongly interact with solvent molecules through various kinds of intermolecular interactions, including hydrogen bonding, ion‐dipole interactions, and vdW forces.^[^ ^29^ ^]^ These ion transport properties play a crucial role in many processes, e.g., biochemistry, batteries and supercapacitors, membrane separation science, microfluidics and nanofluidics, and various other processes.

## 3 Biological Nanoionics

**Figure 3:**
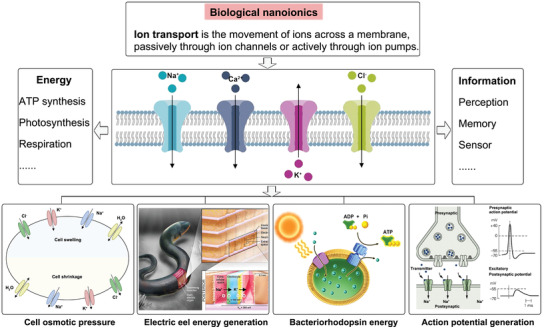
Typical examples of biological nanoionics. Ions are deeply involved in various living processes including energy and information systems like cell osmotic pressure maintenance, electric eel energy generation, solar energy harvesting of bacteriorhodopsin, and action potential generation. In all these processes, controllable ion transport through biological nanopore are recognized as the fundamental step. Reproduced with permission.^[^ ^33^ ^]^ Copyright 2017, Springer Nature.

Precisely controlled ion transport across cells and organelles are crucial for life. In a biological neural system, ion channels are the molecular basis for all the electrical activities in initiation, processing, and transmission of information. In single cells, ion flows are involved in the regulation of a wide range of processes from signaling, action potential, pH balance, volume regulation, to and the cell cycle. In higher forms of living organisms, they also determine immune responses, fertilization, muscle contraction, and secretion (**Figure** **3**).^[^ ^30^ ^]^ For example, the volume of cells is typically tightly and precisely controlled by ion transport to sustain their normal function and survival. In a single cell, volume control involves a wide spectrum of processes, including ion channels on the membrane that permit passive ion exchange between the extracellular fluid and intracellular cytosol, and active ion transporters that pump ions against a concentration gradient.^[^ ^31^ ^]^ Another example is the electric eel (Electrophorus, a genus of neotropical freshwater fish). They can generate high voltages of up to 600 V and currents of 1 A when they are attacked by their natural predators. Na^+^ and K^+^ channels in the cell membrane that open and close periodically are the basis of their specialized electric organs. Benefiting from its relative permeability to Na^+^ and K^+^ ions, a single unit of charge‐generating organ can generate a small action potential of ≈+65 mV, and 1000 units connected in series and in parallel resulting in the simultaneous high voltage and high current.^[^ ^32^, ^33^ ^]^ Some bacteriorhodopsin transforms solar energy into adenosine triphosphate (ATP) by light‐driven ion pumps, which can pump ions (protons) from low to high concentration to build an electrochemical potential gradient which powers the ATPase for ATP synthesis.^[^ ^34^ ^]^ In the mammalian brain, the distribution of calcium ions in dendrites may represent another essential variable for the processing and storing of information. Calcium ions enter the dendrites through voltage‐gated ion channels in the cell membrane, which results in rapid local modulations of calcium concentration within the dendritic tree.^[^ ^35^ ^]^

All of these important energy and information processes rely on precise ion transport through protein nanopores. The protein ion channels that transport ions in and out of cell membranes fall into two general classes: passive conduits named ion channels, through which ions flow down gradients of concentration and electric potential, and ion pumps, which actively push ions against concentration gradients by consuming energy from ATP or other sources.^[^ ^30^ ^]^

Ion channels are the passageways that permit the transport of a single type of ion because ion channels generally have a selectivity filter. In some specific cases, ion channels are possible facilitators of the passage of multiple ion species; for example, some ligand‐gated ion channels permit the transport of Na^+^ and Ca^2+^. The ion flows are not incontinent and are regulated to turn on by the gates of ion channels only when needed. The resulting ionic current, generated by the movement of charged ions through ion channels, is crucial for the realization of many biological functions. Meanwhile, different ion flows have distinctive roles. For example, the ionic currents of Na^+^ or K^+^ ion flows act as physical signals (action potential), whereas the concentrations of Ca^2+^ ions themselves usually represent information. Ionic currents of Cl^−^ ion flow tend to stabilize membrane potentials, such as in skeletal muscle fibers and at inhibitory synapses.

The term ion pump is the collective name of all the protein transporters capable of thermodynamic uphill transport (so‐called active transport). Based on the energy source, ion pumps are divided into two different groups: primary pumps and secondary pumps. In primary ion pumps, the energy comes from various sources, including ATP hydrolysis, oxidation–reduction reactions, and light. Secondary ion pumps (also called co‐transporters or counter‐transporters or exchangers) exploit the energy stored in electrochemical gradients of other ions.

The different behaviors of ion channels and ion pumps – passive, thermodynamic downhill and high‐speed ion movement through channels, versus active, thermodynamic uphill transport, frequent incorporation of enzyme‐like reaction mechanisms, and low‐speed ion movement through pumps – led to notable features to understand them. However, both ion channels and ion pumps have fine structure and specific surface properties, resulting in precisely controlled ion‐transport functions. For example, the Na‐K nonselective channel only allows one fully hydrated Na^+^ ion to enter the selectivity filter; the potassium filter from Streptomyces lividans contains two K^+^ ions ≈7.5 Å apart, with a single water molecule in between; and, in calmodulin, each calcium channel could simultaneously bind two Ca^2+^ ions.^[^ ^36^ ^]^

Herein, we refer to all these biological ion‐transport‐related processes as biological nanoionics in order to distinguish from the artificial and hybrid nanoionics systems discussed in the following sections. These biological ion‐transport‐related energy systems and information processors (biological nanoionics) provide an ideal model to solve existing energy‐ and information‐related modern problems. Mimicking some of their structures and functionalities in artificial devices has unlimited potential, as in designing new energy‐efficient computation architectures based on ion transport rather than electron transport.

## 4 Artificial Nanoionics

Beyond biological systems, artificial ion‐transport devices have become increasingly compact and integrated with the development of nanoscience and nanotechnology. The decrease in the size of different components in a device is usually accompanied by several effects. First, the contribution from interface becomes more significant, whether it is a solid–solid or solid–liquid interface that is related to ion transport. For a solid–solid interface, “nanosize” causes a large increase in the proportion of atoms at the phase boundary, where the electron state and coordination differ from those of the bulk. This results in a quick increase of ion‐diffusion rates and a growth in diffusion flux volume.^[^ ^37^ ^]^ For a solid–liquid interface, various weak interactions, including hydrogen‐bond and vdW forces that are neglected in bulk systems play a crucial role.^[^ ^26^ ^]^ These differences also influence the sorption properties of nanoconfinement and the distribution of charge carriers between the nanostructure and ion‐carrying phase (e.g., electrolyte or ionic liquid). Second, the thermodynamic characteristics of the nanoconfinement objects are amended, which enables new scenarios for the redistribution of charge carriers in nanoconfinement systems. Third, a variation of energy levels will occur at the interface (solid/solid or solid/liquid interface). All of these effects result in the hallmarks of ion transport in nano‐ or sub‐nanosystems which in turn determines the applications of those devices. In this section, solid‐state and liquid nanoionics will be introduced separately based on the different ion‐transport medium.

### 4.1 Artificial Solid‐State Nanoionics

Solid‐state nanoionics is a subfield of solid‐state ionics, which is a subfield of solid‐state chemistry and physics, and in which ion‐transport properties, phenomena, effects, and applications in solid materials, such as ionic crystals, ionic glass, and polyelectrolytes. The study of solid‐state ionics began in the 19th Century, first with ion‐transport in some solid electrolytes, such as Ag_2_S and PbF_2_.^[^ ^38^ ^]^ The ionic‐conductive property of some solid‐state materials originates from the fact that crystals always have defects, which results in vacant sites. The vacant sites will be a suitable position to host an ion that jumps from the immediate vicinity, leaving the previous site of the ion vacant for another ion. This process enables ion transport in solid‐state materials, leading to conductivity.^[^ ^39^ ^]^ Later, solid‐state nanoionics became an important subject because it was found that nanosized systems may promote ionic conductivity in solid‐state materials. For example, after adding nanopowder of “insulating” material (alumina) to lithium iodide (LiI) crystals, the ionic conductivity of the LiI crystals could be increased by 50 times.^[^ ^40^ ^]^ The conductivity of ionic polymers can also be increased using a similar principle.^[^ ^41^ ^]^ These unexpected results were explained by charge separation at the matrix‐nanoparticle interface, which afforded additional conductive channels to the matrix. The area of this interface was increased effectively by adding filler particles of small size, resulting in improved conductivity.^[^ ^42^, ^43^ ^]^

The definition, mechanism, phenomena, application, and essential factors of solid‐state nanoionics have been well summarized in a number of review articles.^[^ ^24^, ^25^, ^44^, ^45^, ^46^, ^47^, ^48^ ^]^ Here, we briefly introduce solid‐state nanoionics and highlight its applications in energy and information systems.

**Figure 4:**
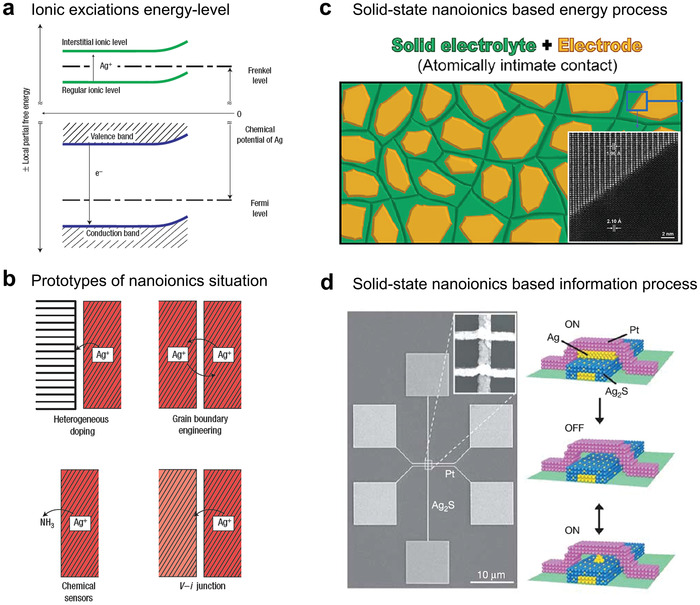
Concept of solid‐state nanoionics and its application in energy and information processes. a) The ionic excitations can be represented in a (partial free) energy‐level diagram, as is commonly used for electronic excitations. b) Four prototype situations involving boundaries to solid ion conductors (using Ag^+^ conductor as an example): Contact to an insulating phase that adsorbs Ag^+^; grain boundary; contact to an acid–base active gas phase; contact to a second Ag^+^ conductor. Reproduced with permission.^[^ ^25^ ^]^ Copyright 2005, Springer Nature. c) Atomically intimate solid–solid interface to improve ion transport in solid electrolyte battery. Reproduced with permission.^[^ ^53^ ^]^ Copyright 2019, Elsevier. d) Ion transport in solid‐state materials for ionic memristor. Reproduced with permission.^[^ ^54^ ^]^ Copyright 2005, Springer Nature.

Before discussing its applications, we must clarify how an interface influences the ion transport properties in a solid system. First, we must clarify that the noncontinuum region of different structures, including the transition zone between two differently oriented grains or the interface between two different phases, is the core of an interface. Second, the respective electrochemical potential for each ion must be constant throughout the sample in equilibrium and is identical in each building element. Hence, ion redistribution will be caused by the structural variation at the interface, which can be reflected by the energy‐level bending of ions and electrons (**Figure** **4**a). Figure 4b summarizes several examples of ionic charge‐transfer effects at equilibrium contacts (using Ag^+^ conductor as an example), including ionic conductor/insulator heterogeneous doping, grain‐boundary engineering, ionic conductor/active gas doping, and ionic conductor A/ionic conductor B heterogeneous doping.

In general, decreasing filler‐particle sizes to the nanometer scale suggests a substantial increase of the proportion of interfaces, resulting in an increased impact of the interfacial properties on the overall materials property (trivial size effects).^[^ ^44^ ^]^ This means that increasing the density of interfaces will result in the overlap of space‐charge zones and ionic confinement effects. This can lead to completely new properties, e.g., allowing for lithium storage in a composite in which the individual phases are not electrochemically active. Beyond the enhanced effective conductivity, the benefit of nanosized interfaces can also realize rapid bulk storage because of the reduction of the effective diffusion length (Figure 4c). This means that ion storage in nanoconfinement systems can be extraordinarily fast because the diffusion equilibration time is proportional to the square of the diffusion length. By calculation, a size reduction from 1 to 10 nm reduces the equilibration time by 10 orders of magnitude. Therefore, nanoionics is increasingly considered to be useful in energy conversion and storage, information storage and transduction, as well as reaction kinetics and catalysis.^[^ ^49^, ^50^ ^]^

For electrochemical energy storage, such as nonaqueous Li–O_2_ batteries, fast charging and discharging are considered as a major factor limiting its practical application. One of the solution is nanoionic, i.e., by fabricating nanostructure materials to increase the ion‐transport kinetics. By constructing a mixed system of nanocrystalline ion conductors and nanocrystalline insulators, Heitjans et al.^[^ ^51^, ^52^ ^]^ found that the Li‐ion diffusivity increased effectively, which can be confirmed by ^[^ ^7^ ^]^ Li nuclear‐magnetic‐resonance line‐shape analyses and spin‐lattice relaxation measurements. Meanwhile, the authors also found that various defects and strain introduced by high‐energy ball milling can also promote ion transport. More interestingly, it was also shown that slow ions are located inside the grains while fast ions are at the interfacial regions because of the interface energy‐level bending.^[^ ^52^ ^]^ Recently, Li et al.^[^ ^53^ ^]^ reported an atomically intimate solid–solid contact by forming epitaxial interfaces, which can significantly improve ion conductivity. All these examples confirmed the importance of nanoionics in energy conversion and storage.

The same principle is suitable for solid‐state ionic information storage and transduction, e.g., for ion‐driven resistive switching (ionic memristor) based on ionic processes in solid‐state thin films. As a next generation of memristor, ion‐driven resistive‐switching devices have been widely studied because of their remarkable performance in terms of switching speed, retention, endurance, and scalability.^[^ ^46^, ^54^ ^]^ In a memristor device, a dielectric thin film is typically sandwiched between two electrodes. The conductivity (or resistance) of the device can be reversibly modulated by applying an external voltage, resulting in a resistive‐switching effect. In ionic resistive‐switching devices, the adjustable conductivity originates from the rearrangement of atoms/ions in the dielectric thin film through ionic drift/diffusion and electrochemical processes that lead to the formation/rupture of nanoscale conductive paths (filaments) between the two electrodes. Therefore, the design and implementation of ionic memristor devices with improved performance and reliability depends on the possibility to control these internal ionic processes. For example, it is possible to fabricate an atomic‐scale ionic switch by controlling the reconfiguration of silver atoms within an atomic‐scale junction (Figure 4d).^[^ ^55^ ^]^ Such “atomic relays” operate at room temperature, and the only movable parts of the switch are the contacting atoms, which acts as a nanometer‐scale gate.

Additionally, recent studies showed that more complicated biological effects that are critical for learning and memory can also be emulated by the internal ionic processes during resistive switching, e.g., nonvolatile memories, including resistive random‐access memory, conducting bridge random‐access memory, and phase‐change memory. This enables efficient neuromorphic systems to be implemented using solid‐state devices and networks.^[^ ^4^, ^56^ ^]^ Meanwhile, controlled ionic processes can also be used to directly modify the composition and/or structure of the material itself at the atomic scale, allowing new nanostructures to be built on the fly, in a reconfigurable fashion.

### 4.2 Artificial Liquid Nanoionics

Liquid nanoionics refers to ion transport in liquid phase that is confined in nanoscale systems, e.g., in nanochannels, nanopores, nanotubes, or porous materials.^[^ ^6^, ^17^ ^]^ In a liquid medium, ions exist as solvent‐free ionic liquids or solvated ions, i.e., ions surrounded by solvent molecules. Different from ion diffusion in bulk systems, ions in nanospace demonstrate very different behaviors. Spatial confinement could constrain the motion of ions and/or modify their interactions with each other and with the nanostructure surface, resulting in some anomalous ion‐transport properties.

Several factors including pore size distribution, pore conductivity, pore connectivity, charging dynamics, and ion concentration will affect ionic diffusion or the flow of ions in and out of the pores.^[^ ^57^, ^58^, ^59^, ^60^, ^61^, ^62^ ^]^ Among them, pore size is the most important factor. In this section, ion transport in nanoscale (2–100 nm) and microporous (typical below 2 nm) will be introduced separately due to their different interaction forces. Meanwhile, the effects of other factors including pore conductivity, charging dynamics and pore connectivity will also be introduced briefly.

#### 4.2.1 Ion Transport Mechanisms in Nanoconfinement

**Figure 5:**
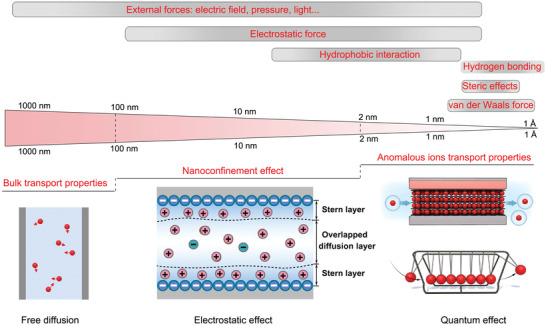
Scales of interactions for ions in liquid nanoionics system. In the systems with diameter larger than 100 nm, ions exhibit bulk free diffusion property. In the regime between 2 and 100 nm, electric double layer controlled ion transport domains. Decreasing the size below to 2 nm, some anomalous ion transport (quantum effect) properties are revealed. Scales of interactions for ions in the top of the figure: Reproduced with permission.^[^ ^28^ ^]^ Copyright 2019, Elsevier. Newton's cradle model in the bottom right corner: Reproduced with permission.^[^ ^69^ ^]^ Copyright 2021, AAAS.

**Figure** **5** shows the interactions, including hydrophobic force, vdW forces, and electrostatic attraction, when ions are confined in a nanospace. For materials of various sizes, the dominant ionic interactions may vary. It should be noted that each interaction is effective only at certain length scales, generally less than 100 nm. The interplay of these different interactions will significantly influence how ions are distributed in the nanospace and how they transport through a nanochannel. Taking the solid/liquid interface as an example, ion redistribution in the presence of electrical charge/bias will result in an electrical double layer (EDL), the thickness of which is defined as the Debye length.^[^ ^63^, ^64^, ^65^ ^]^ The Debye screening length is inversely proportional to the square root of the ionic strength, and it is generally in the range of 100 nm. However, for solvent‐free room‐temperature ionic liquids, the Debye screening length is very small and the Debye theory does not apply. The phenomena could not simply be explained in terms of Debye screening, and a more elaborate theory must be used, which will be introduced later.

When the size of the nanospace is comparable to the Debye length, solvated ions in nanoconfinement will “feel” the electrostatic forces from the adjacent surface, resulting in regulatable ion‐transport properties. When the dimension of nanopores is further decreased to a comparable size of solvated ions (in general, smaller than 1 nm),^[^ ^66^ ^]^ interactions between solvent molecules and ions could be affected by the neighboring ions or the nanospace surface, resulting in the restructuring of solvated ions. In general, the restructured solvation shells around ions in nanospace would be thinner than those in bulk.^[^ ^67^ ^]^ In this case, the average inter‐ion distance could then be reduced. If the interion distance is comparable to or smaller than dimensions of the Bjerrum length (a term used to characterize the distance between two charges in a medium with a dielectric constant in which the Coulombic energy is equal to the thermal energy ^[^ ^68^ ^]^), the weak interion interactions, including hydrogen‐bonding and vdW interactions, become dominant. In this case, ion‐transport behavior becomes more complex and includes a range of unexpected solvation‐involved nanoionic phenomena, e.g., ultrafast ion transport.^[^ ^69^ ^]^

However, ion transport properties will be different in the case of conducting confinements. It is clear that the ion‐ion interaction follows the Coulomb law in the case of nonconducting confinements. While in a conducting confinement, the interaction energy between two ions decays exponentially because of the screening of electrostatic interactions induced by the electrons of the conducting walls.^[^ ^70^, ^71^, ^72^ ^]^ This will result in several interesting nanoionic phenomena, e.g., superionic states. More specifically, ion diffusion will be highly modified in subnanometer pores as the pore becomes charged. The self‐diffusion coefficient of ions confined in subnanometer pores will exhibit a bell‐shaped curve as charging proceeds: At the potential of zero charge, ions inside neutral subnanometer pores diffuse very slowly; as charging proceeds, the self‐diffusion of ions greatly accelerates, even becoming a few times faster than that in bulk room temperature ionic liquids; their diffusion finally slows down when the pore becomes strongly charged.^[^ ^59^, ^71^, ^73^ ^]^ These results also highlight the importance of precisely controlling pore size to realize fast ion diffusion in charged pores. Beyond that, it is suggested that pore connectivity, the distribution of pore throats and the charging dynamics are also important characteristics to consider when improving the ion diffusion and optimizing the existing and developing new porous electrode materials for supercapacitor.^[^ ^57^, ^73^, ^74^ ^]^

#### 4.2.2 Ion Transport Phenomenon in Large Pore Size (2–100 nm)

**Figure 6:**
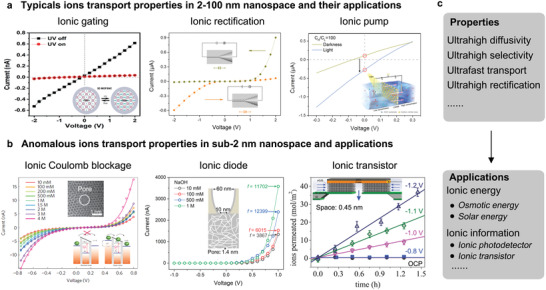
Typical examples of ion transport properties in scale of 2–100 nm and below 2 nm. a) In scale of 2–100 nm, ions can be manipulated to transport in specific direction, and exhibit properties of ionic gating, ionic rectification, and ionic pump. From left to right: Reproduced with permission.^[^ ^83^ ^]^ Copyright 2020, Wiley‐VCH. Reproduced with permission.^[^ ^79^ ^]^ Copyright 2016, Wiley‐VCH. Reproduced with permission.^[^ ^80^ ^]^ Copyright 2019, Springer Nature. b) In scale below 2 nm, some quantum‐mechanical‐like features are confirmed, for example ionic Coulomb blockage, ionic diode, and ionic transistor. From left to right: Reproduced with permission.^[^ ^121^ ^]^ Copyright 2016, Springer Nature. Reproduced with permission.^[^ ^124^ ^]^ Copyright 2021, Springer Nature. Reproduced with permission.^[^ ^69^ ^]^ Copyright 2021, AAAS. c) The fundamental properties of liquid nanoionics and its applications in energy and information processors.

In conventional nanopores of larger sizes (2–100 nm), several factors, including external force, electrostatic force between ions and interface, wettability, and steric effect, will affect the ion‐transport properties. Ion transport exhibits well‐organized and controllable properties, e.g., ionic gating,^[^ ^75^, ^76^, ^77^ ^]^ ionic rectification,^[^ ^78^, ^79^ ^]^ and ion pump,^[^ ^80^, ^81^ ^]^ which are also found in biological nanopores (**Figure** **6**a).

Ionic gating means that the nanofluidic system can work as a gate for ion flow, and it can be opened by stimuli,^[^ ^82^ ^]^ such as voltage‐gated biological nanopores. For example, Qian et al.^[^ ^83^ ^]^ reported a responsive ionic gate with a high on/off ratio up to 17.8 based on azobenzene (Azo)‐molecule‐modified metal–organic framework nanochannels. Azo is one of the most popular photoresponsive organic groups, as an Azo group can undergo reversible isomerization between trans and cis when triggered by ultraviolet irradiation or heat, leading to an unusual open‐to‐closed switching of the channel. Beyond that, many external stimuli, including electric,^[^ ^84^, ^85^ ^]^ pH,^[^ ^86^ ^]^ thermal,^[^ ^87^ ^]^ magnetism,^[^ ^88^ ^]^ specific molecules and ions,^[^ ^89^, ^90^, ^91^ ^]^ also can be used as a trigger to activate the ionic gate.

Ionic rectification is the phenomenon where the ion flow in one direction is suppressed while it is unimpeded in another direction.^[^ ^92^ ^]^ This is one of the most common ion‐transport properties of biological nanopores, and it has been replicated and studied sufficiently by artificial systems. Xiao et al.^[^ ^79^ ^]^ reported an ionic rectification system with a rectification ratio larger than 500 by constructing an asymmetric funnel‐shaped system. They modified the cylindrical and conical segments of a funnel‐shaped nanochannel with different molecules, leading to synergic asymmetric structure and asymmetric surface charge distribution. To realize ionic rectification in nanofluidic systems, an asymmetric configuration is crucial, whether asymmetric structure or asymmetric surface properties (wettability or surface charge density) exist.^[^ ^75^, ^93^ ^]^ Based on this principle, Hou et al.^[^ ^94^ ^]^ reported a dynamic curvature nanochannel system with a reversible rectification switch that endows the artificial system with flexible ion‐transport properties. The nanofluidic system with an ionic rectification property can also be used for the unidirectional transport of several charged drugs or cell messengers, which can then be used for smart drug delivery or to study cell commination by avoiding diffusive leakages.^[^ ^95^ ^]^

Ion pumping means that ions can transport against a concentration gradient by consuming external energy. In biological systems, ion pumping is used to pump protons from low to high concentration to create an electrochemical potential difference, which can be then used to drive an ATPase to produce ATP.^[^ ^30^ ^]^ To realize the ion‐pumping function in artificial systems, some photo‐responsive molecules are introduced into a lipid membrane or porous membrane.^[^ ^96^, ^97^, ^98^, ^99^, ^100^ ^]^ Furthermore, nanofluidic‐based, light‐driven ion‐transport systems can work based on three different underlying working principles, i.e., photochemical, photoelectric, and photothermal effects, by creating an asymmetric surface charge distribution.^[^ ^101^ ^]^ For example, some semiconductor nanostructures can realize the ion‐pumping function directly via ion transport coupled by photoinduced carriers. Xiao et al.^[^ ^80^ ^]^ reported a carbon nitride nanotube membrane that can achieve pumped ion transport against a 5000 concentration gradient by creating a gradient surface charge density with light illumination.

These controllable ion‐transport properties in an artificial nanochannel of size between 2 and 100 nm are associated with many ion‐related applications, such as osmosis energy harvesting,^[^ ^28^, ^102^ ^]^ water desalination,^[^ ^103^ ^]^ and various ionic sensors,^[^ ^104^ ^]^ and they have been summarized well by many reviewers.^[^ ^5^, ^17^, ^105^, ^106^ ^]^

#### 4.2.3 Ion Transport Phenomenon in Small Pore Size (<2 nm)

With decreasing pore diameter, weak interaction forces, including vdW and hydrogen‐bonding interactions, play an increasingly important role in ion transport, resulting in anomalous ion‐transport properties and applications (Figure 6b).^[^ ^107^, ^108^, ^109^ ^]^ This phenomenon is especially obvious in the study of electrical double‐layer capacitors (EDLCs). It has been shown that a decrease in the average pore size of carbonaceous materials used in EDLCs can generate an increase in the capacitance of the cell, especially when the size of the pores is commensurate with that of the bare ions of the electrolyte.^[^ ^72^, ^110^, ^111^ ^]^

A different explanation has been given in cases of solvent‐free ionic liquids and solvated ions in aqueous solution. For confined ionic liquids, the non‐Coulombic structure formation is made possible by the repulsive electrostatic interactions between co‐ions caused by image charges induced in the carbon walls, resulting in the superionic state.^[^ ^112^ ^]^ For example, Gogotsi and Simon^[^ ^72^, ^113^ ^]^ found that porous carbon with pores smaller than the size of ions will lead to anomalous capacitance, which then be explained as the superionic state by Kornyshev and co‐workers.^[^ ^114^, ^115^ ^]^ Later, Kaneko and co‐workers ^[^ ^116^ ^]^ showed that Coulombic ordering is reduced when the pores can accommodate only a single layer of ions, which can confirm that ions in such tiny pores are in a superionic state. Some other work tried to explain the anomalous capacitance from the view of energy barriers. For example, Bruin and co‐workers ^[^ ^112^ ^]^ explained that adsorption energies gradually increase with decreasing pore size and show a maximum when the pore size is slightly greater than the dimensions of the adsorbed ion and the attractive vdW forces dominate the interaction. At smaller pore diameters, the adsorption energy sharply declines and becomes repulsive as a result of geometry deformations of the ion. This phenomenon is particularly noteworthy in ionophobic porous materials.^[^ ^114^ ^]^ By overcoming the energy barrier of ionophobic porous materials, it is possible to enhance the charging dynamics and improve energy and power densities of supercapacitors.^[^ ^117^ ^]^

In the case of ions with a solvation shell, the mechanism is slightly different. The hydrated ions must undergo the dehydration process or deformation to be squashed into the channel.^[^ ^118^, ^119^, ^120^ ^]^ This suggests the establishment of an intrinsic energy barrier that forbids ion entry into the channel.^[^ ^112^ ^]^ However, the energy barrier can be overcome by different external forces, e.g., an external electric field or increased surface charge density induced by illumination.

Feng et al.,^[^ ^121^ ^]^ reported an ionic Coulomb blockade phenomenon observed in a single‐layer MoS_2_ membrane. In their work, the nanopore used was ≈0.6 nm in diameter, slightly smaller than the hydrated alkali‐metal ions. Therefore, the ions must be dehydrated to pass through the pore, resulting in an additional energy barrier that can be overcome by a larger external electrical field. In this case, the current is suppressed at small voltages and starts to increase sharply when the bias voltage exceeds the threshold value. The observation of this ionic Coulomb blockade phenomenon displays quantum‐mechanical‐like features dominating ionic transport, and it will also offer a new platform to explore novel physics in the research areas of both artificial and biological nanoionics.^[^ ^122^, ^123^ ^]^

The sub‐nanoscale channels also exhibit anomalous unidirectional ion transport (ionic rectification) if their structure is asymmetric. We introduced ionic rectification in the preceding section, stressing that it is an important ion‐transport property of protein channels and can be realized by creating asymmetric elements. If the pore diameter is smaller than 2 nm, the energy barrier will play an important role in determining the rectification ratio. Xiao et al.,^[^ ^124^ ^]^ reported a hierarchical pore architecture of the carbon membrane with a pore size gradient changing from 60 to 1.4 nm, which enables high ionic rectification ratios up to 10^4^ in different environments, including high‐concentration neutral (3 m KCl), acidic (1 m HCl), and alkaline (1 m NaOH) electrolytes. The ultrahigh and stable ionic rectification ratio resulting from the different energy barriers when ion transport in different directions benefits from an asymmetric structure. Similar anomalous unidirectional ion transport was also observed in a MOF membrane.^[^ ^125^ ^]^

Xue et al.,^[^ ^69^ ^]^ fabricated an atomic‐scale ion transistor using a single flake of reduced graphene oxide with channel sizes being ≈3 Å. Because the channel size is smaller than the hydration diameters of alkali‐metal ions, an intrinsic energy barrier is created that forbids ion entry into the channel. Subsequently, a gate voltage was applied to increase the electric charge density of the walls to change the energy barrier, and ultrafast ion transport, namely, one that is 2 orders of magnitude faster than ion diffusion in bulk water, was observed beyond a threshold voltage. The authors also studied permeation rates of different alkali‐metal ions, in which K^+^ > Cs^+^ > Li^+^, which resembles the selection sequence of biological potassium channels. These phenomena can be explained by different electrophoretic mobilities, meaning that ionic species with different charge‐to‐size ratios spend different times in the channel because the enormous electric fields inside EDLs cause transverse ion distributions that yield charge‐dependent mean ion speeds in the flow.^[^ ^126^ ^]^

It can be summarized from the above examples that there is an energy barrier for ion transport when the nanosystem has a diameter smaller than 2 nm.^[^ ^124^, ^125^ ^]^ In terms of this energy barrier's origin, it can be confirmed that ultimate confinement largely reduces the electrostatic iterations because of the existence of the superionic state.^[^ ^116^ ^]^ Several other simulations also suggested that the dielectric constant of aqueous solutions is reduced for very narrow confinements,^[^ ^127^, ^128^, ^129^ ^]^ resulting in strong Coulombic interactions among adjacent ions and promoting their concerted movement with decreasing energy barrier. Of course, the detailed mechanism of ions diffusion in such tiny pore is still not clear and remains open to further simulations and experimental investigation.^[^ ^61^, ^130^ ^]^ However, it is for sure that ions in such a tiny space show different packing states within larger pores or bulk systems, and exhibit anomalous ion‐transport properties, including superfast transport speed and superhigh selectivity (Figure 6c).^[^ ^131^ ^]^ Meanwhile, potential applications are also expected based on ion transport in sub‐nanoconfinement, for example, a decrease in the average pore size of electrode materials used in EDL capacitors will generate an increase in the capacitance of the cell.^[^ ^72^, ^110^ ^]^

## 5 Biological–Artificial Hybrid Nanoionics

As introduced above in the discussion of biological and artificial nanoionics, ions as transport carriers can be used to design various energy and information processes both in vivo or in vitro. The question now becomes “Is it possible to fabricate biological–artificial hybrid nanoionics‐based energy and information systems?” Human–machine interaction (HMI) is a typical biological–artificial information‐exchange system considering that most HMIs are based on electron transport.^[^ ^132^, ^133^ ^]^ However, nervous cells in biological systems communicate with each other by ions and chemical molecules (neurotransmitters), e.g., the generation and transmission of action potential.^[^ ^134^ ^]^ Therefore, it is necessary to develop biological–artificial hybrid nanoionics that work on the principle of ion transport. In this case, it is possible to realize direct neuromodulation without electrical to ionic transduction.

Most of the existing electronic platforms act like passive devices; that is, they are able to monitor cellular activity without providing significant feedback.^[^ ^135^, ^136^ ^]^ However, establishing an active interaction with biological tissues allows a transition from mere recording of biological functions to dynamic interactive control. What is required at this stage is the capability not only to receive biologically encoded inputs to be processed, but also to generate outputs, hence closing the artificial neural circuit.

**Figure 7:**
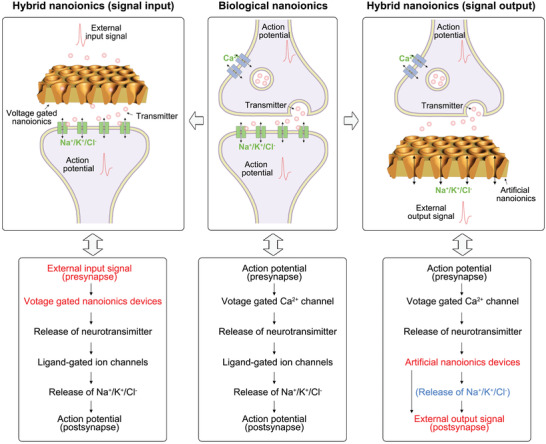
Diagram of biological action potential generation in a neuronal network and bio‐inspired (biological–artificial) hybrid nanoionics. In biological system, the generation of action potential in synapse origins from the ion transport triggered by neurotransmitter. In hybrid nanoionics system, external input signal from artificial nanoionics system can be write as action potential in post‐synapse, or action potential from pre‐synapse can be read by artificial nanoionics devices.

In this case, two different biological–artificial hybrid nanoionics systems are proposed (**Figure** **7**): one for signal input and the other for signal output. For the first case, the input signal is from external stimuli, e.g., light, heat, electricity, pressure, and specific ions or molecules, corresponding to a biological perception system. These external signals can trigger some artificial nanoionics devices, resulting in the release of some specific ions or molecules (neurotransmitters). For example, a voltage‐gated artificial ionic gating can open the gate for mass transport with the voltage above threshold. If the released neurotransmitter (e.g., dopamine) can trigger biological nanopores, Na^+^ or K^+^ ions will flow through the transmembrane to generate an action potential post‐synapse. In this way, a biological–artificial hybrid nanoionics system that works in a way similar to that of its biological counterpart is fabricated. Analogously, biological signals can also be transduced to artificial signals by biological–artificial hybrid nanoionics. These two cases correspond to the “read” (signal‐output model) and “write” (signal‐input model) of biological signals. We can assume that an ionic device integrating these two functions can realize actual human–machine “two‐way” communication.

**Figure 8:**
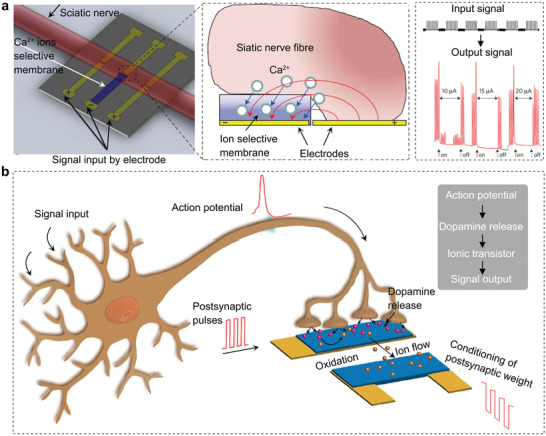
Examples of hybrid nanoionics. a) Artificial signal is transformed to biological signal by ions selective membrane. Reproduced with permission.^[^ ^137^ ^]^ Copyright 2011, Springer Nature. b) Biological action potential is transformed to artificial signal by ionic transistor. Reproduced with permission.^[^ ^9^ ^]^ Copyright 2020, Springer Nature.

This perspective is not idealistic. Some results from proactive research have proved that biological–artificial hybrid nanoionics can realize signal transduction between biological and artificial systems; for example, electrical signals can be transduced to ionic signals (electrically modulating ion concentrations in situ along a nerve) by ion‐selective membranes to activate and inhibit a nerve (**Figure** **8**a).^[^ ^137^ ^]^ Organic electronic ion pumps, another artificial nanoionics device made using the ionic conductor polymer PEDOT:PSS, were widely used to regulate ion or molecule flow for stimulation of individual cells or plants.^[^ ^138^, ^139^, ^140^ ^]^ These hybrid nanoionics move a significant step forward in biology‐technology interfacing by translating electronic addressing signals, through ionic or molecular signaling, into brain‐stem responses. However, the translation should not be limited to electric signals since other external stimuli, such as light, pressure, and heat, also have the possibility to be translated into ionic signals by integrating artificial nanoionics devices into biological systems. In addition, biological signals can also be understood by hybrid nanoionics. Keene et al.^[^ ^9^ ^]^ reported a hybrid synapse by coupling an organic neuromorphic device (artificial ionic transistor) to dopaminergic cells (biological nanoionics), which can then realize neurotransmitter‐mediated synaptic plasticity (Figure 8b). In that work, dopamine exocytosed by dopaminergic cells at the presynaptic end is locally oxidized at the postsynaptic gate electrode, resulting in the generation of external output signals.

We envision that by combining “input” and “output” biological–artificial hybrid nanoionics systems that can not only signal “read” and “write,” but a chemical communication between neurons can also be introduced or repaired.

**Figure 9:**
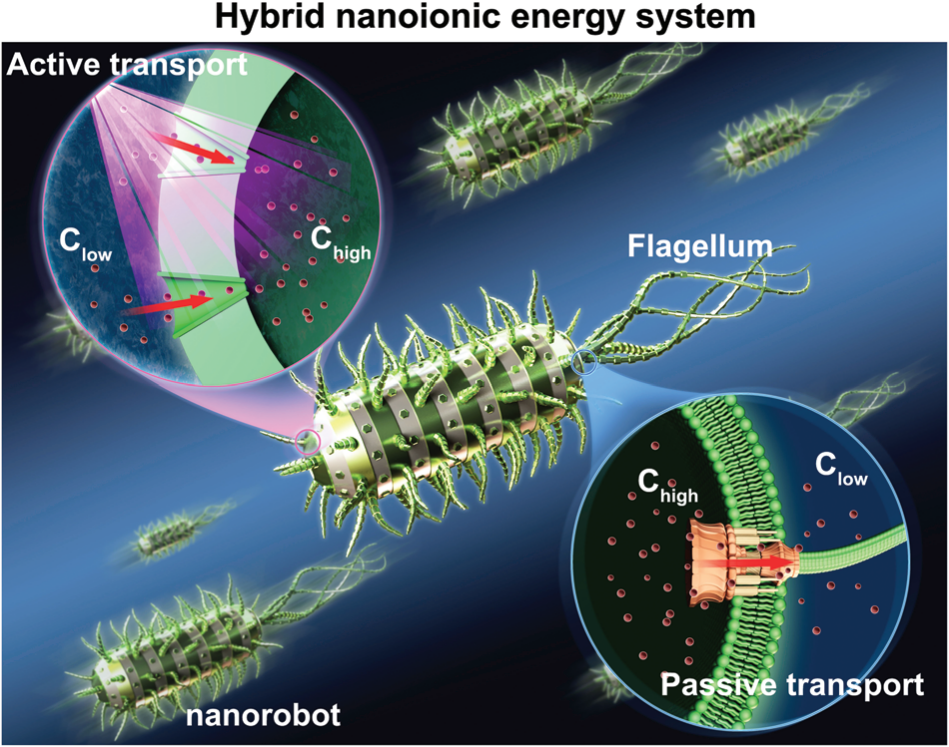
Diagram of hybrid nanoionics energy system by integrating light‐driven ion pump and biological molecule motor. Reproduced with permission.^[^ ^28^ ^]^ Copyright 2019, Elsevier.

Unlike the attractive biological–artificial hybrid nanoionics information processes, hybrid energy processes are largely ignored because all energy‐generation processes emphasize conversion efficiency and energy density, both of which are drawbacks of ion‐based systems because of the speed of slow‐moving ions. We must hypothesize, however, that electron‐based energy systems cannot be used everywhere, e.g., the sustaining and biocompatible energy source for nanorobots in vivo (**Figure** **9**). In this case, it is worth constructing some biological–artificial hybrid nanoionics energy systems that work to generate biological nanoionics energy. Enlightened by biological ATP synthesis process in vivo, a hybrid nanoionics energy system that can drive ion transport directionally to build a concentration gradient for ATP synthesis by a molecule motor is possible.^[^ ^141^ ^]^ Meanwhile, as stated above, the traditional idea that ions move slowly has been challenged in several recently published works, especially in sub‐nanoscale space.^[^ ^124^ ^]^ In such a small space, superfast ion transport is possible, e.g., by Newton's ionic cradle model.^[^ ^142^ ^]^ Therefore, the energy‐conversion efficiency and energy density of ionic energy systems could be improved. However, these concepts must be verified before they can be used to solve materials and nanostructure problems.

## 6 Prospects and Outlook: Why Nanoionics?

In view of the “omnipotence” of nanoelectronics, a question arises, namely, “Why do we need nanoionics?” Before answering, we must clarify that nanoelectronics is not an isolated scientific development process. Nanophotonics,^[^ ^143^ ^]^ moletronics,^[^ ^144^ ^]^ and nanoionics ^[^ ^25^ ^]^ must always complement it, even though their value to the process is more or less eclipsed by nanoelectronics. As semiconductor technology moves ever closer to the ultimate physical limits for scaling of devices that utilize electrons, several new options based on working principles other than electron transport should be developed. Therefore, nanoionics has the potential to provide the solution for this challenge.

**Figure 10:**
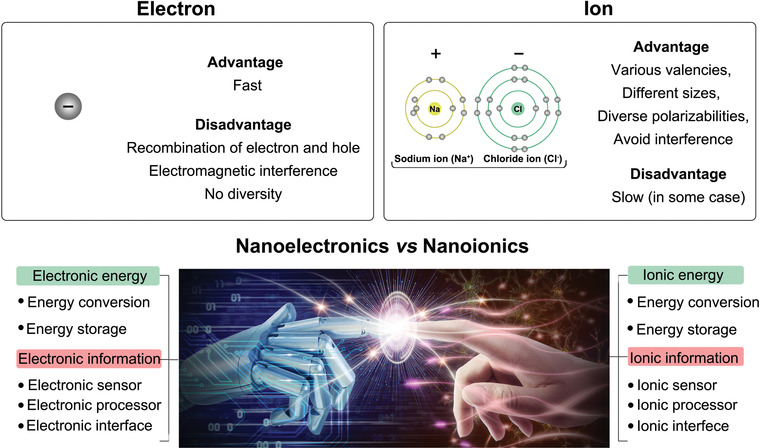
Compared to electrons, ions have many unique advantages to design energy and information devices, for example various valencies, different sizes and diverse polarizabilities. These characterizations enable all energy and information processors that constructed by electrons can be redefined by ions.

A simple answer to “Why nanoionics?” is that Nature chooses ions to sustain vital functions of the human body. On the bottom level of biological intelligence, different types of ion channels form the molecular basis for electrical activities in neurons and support the transmission and processing of information. While Nature has always served as a great inspiration to develop methods for achieving lower‐power and real‐time artificial intelligence systems, it is obvious that nanoionics holds great potential in the development of ionic information processors (**Figure** **10**). Despite their slow speed, ions have several remarkable advantages over electrons, e.g., relative insensibility to electric field noise, and they are heavy enough to avoid electric or magnetic noise. Ions that bear the same unit charge, in general, are at least 1000 times heavier than their electron counterparts. In this case, the electric or magnetic field must be much stronger to significantly interfere with charge flow in nanoionics. Moreover, a counterintuitive suggestion is that a heavier‐mass information carrier is preferred for smaller devices at sub‐2 nm scales, although a common‐sense observation is that lighter mass moves faster and requires less energy.^[^ ^145^ ^]^ Another remarkable advantage for heavy carriers is that lighter mass in smaller devices has a limited information‐storage time because of quantum mechanical tunneling, while the use of larger mass will decrease tunneling probability. Meanwhile, ions have a smell and color (that is, various valences, sizes, polarizabilities, etc.) and one could harness these supplementary signatures to design more efficient information processors.^[^ ^17^ ^]^ For example, Robin et al.^[^ ^19^ ^]^ used theory and simulations to predict that 2D nanofluidic channels can show nonlinear conduction and function as memory‐effect transistors. By incorporating two nanofluidic memristors in an elementary circuit that refers to Hodgkin's and Huxley's model,^[^ ^146^ ^]^ the neuromorphic responses of emitting voltage spikes were reproduced in simulations of experimental devices. Other advantages including low power consumption and less heat generation, as well as relative insensibility to electric field noise, can also be expected from nanoionics circuitry.

Regarding ionic energy, solid‐state nanoionics has shown its broad applications, e.g., in solid electrolyte batteries.^[^ ^147^ ^]^ By decreasing the size to nanoscale, a high density of interfaces (including grain and phase boundaries as well as free surfaces), which are structurally different from bulk, are obtained in nanostructured materials and can improve the crucial electrical performance of batteries. For example, nanocrystalline electrodes exhibit both high theoretical capacity and high Coulombic efficiency.^[^ ^14^ ^]^ The development of liquid nanoionics for energy generation seems slightly slow because of its low efficiency, which is at present approximately a few percents,^[^ ^105^ ^]^ compared with efficiencies of up to ≈95% for standard rotational electromagnetic generators.^[^ ^148^ ^]^ Whether the well‐developed reverse electrodialysis (RED) ^[^ ^149^, ^150^ ^]^ or recently developed nanofluidic RED,^[^ ^28^, ^105^ ^]^ their energy density and efficiency are not ideal enough for large‐scale commercial applications due to the instinctive drawback of slow ion‐transport speed. Increasingly more strategies are proposed to increase efficiency and implement parallelization in recent years. One way to achieve both purpose is to improve ion‐transport speed and efficiency by integrating multiple external energy sources, e.g., light or thermal energy and concentration gradient.^[^ ^151^, ^152^ ^]^ Another way is to construct a series or parallel connected circuit; for example, Schroeder et al. reported a nanoionic energy system that can generate a high voltage of 110 V at open circuit by connecting thousands of ionic conductive gel compartments in series.^[^ ^33^ ^]^ Therefore, it is expected that all electron‐transport‐based energy and information processors can be soon redefined by nanoionics.

Since nanoionics is an up‐and‐coming research field, it has been foreseen that many difficulties must be solved before its real translation. For nanoionics systems, nanoscale structure and ion‐transport properties are two important factors. What's more complicated is how to integrate these two factors into one system. Therefore, finding candidate materials that are suitable for nanofabrication will be the first challenge. Regarding solid‐state ion‐transport materials, construction of the nanostructure is required. Furthermore, it is difficult to process liquid or semisolid systems, e.g., ionic hydrogels, at nanoscale. Although some ionic interfaces or ionic cables have been reported to communicate with biological systems, ions used in such implementations are not as controllable as their biological counterparts without nanostructures. Random ion transport can only be ordered by an external electric field. In this case, “armor” is needed, e.g., 1D nanochannels and nanotubes, 2D layered space, or 3D porous structure, which can work as a template for these liquid systems or semisolid materials, thus enabling ion transport with precise controllability.

Overall, nanoionics refers to a new area of research in which ion transport is conducted on a nanoconfined space. It may lead to innovative technologies one day by utilizing ions as conductive carriers. Such new methods and technologies could mimic many biological functions, even if they do not have the exact same mechanisms of biological intelligence. This, in turn, may facilitate manufacturing of nanoionic devices for use in science and even in daily life in the future, which may have significant advantages over some of today's electronic technologies. An evident example is the brain, which is a natural ionic system that still exceeds the capabilities of electronic processors in many tasks. However, we must admit that nanoionic devices are not expected to be faster than electronic ones owing to their slow ion‐transport speed. However, they may be able to carry out high‐level information processing such as writing and erasing information under the condition of low energy consumption. One of their potential applications is to construct nanoionics‐based computer memory devices that are much longer‐lasting than the electronic ones in use today. Such an application will be essential for safe, long‐term storage of information. Meanwhile, similar to the application in information and sensory technology, it is expected that nanoionics will play an important a role in energy conversion and storage, e.g., in supercapacitors.

Nanoionics research is in its infancy and its potential is unlimited. Undoubtedly, many challenges have yet to be overcome. Nevertheless, given the rapid technological advancement in nanoscience, as well as the exceptional potential benefits, nanoionics research deserves exploration in the near future. Exploiting the full power of nanoionics to fabricate active devices that are currently dominated by nanoelectronics will lead to the invention of new artificial intelligence forms that are crucially needed and have the potential to be world changing.

## Conflict of Interest

The authors declare no conflict of interest.
